# New Horizons in Research on Diabetic Complications of the Eye: Special Emphasis on Diabetic Cataracts and Retinopathy

**DOI:** 10.1155/2010/979040

**Published:** 2010-10-20

**Authors:** Kavita R. Hegde, Renu A. Kowluru, Susanne Mohr, Ram H. Nagaraj, J. Mark Petrash

**Affiliations:** ^1^Department of Natural Sciences, Coppin State University, Baltimore, MD 21216, USA; ^2^Departments of Ophthalmology, Anatomy and Cell Biology, Kresge Eye Institute, Wayne State University School of Medicine, Detroit, MI 48201, USA; ^3^Department of Physiology, Michigan State University, East Lansing, MI 48824, USA; ^4^Department of Ophthalmology and Visual Sciences, School of Medicine, Case Western Reserve University, Cleveland, OH 44106, USA; ^5^Department of Ophthalmology, Rocky Mountain Lions Eye Institute, University of Colorado Denver, Aurora, CO 80111, USA

The incidence of diabetes is increasing at an alarming rate all over the world. As a consequence, the complications related to diabetes, including those affecting the eye (cataracts and retinopathy), nerve, and kidneys, are also becoming more prevalent. In the eye, diabetes affects both lens and retina resulting in devastating effects on vision. Although significant advances have been made towards understanding their pathophysiology using animal models of diabetes, progress in the area of development of pharmacological means for their prevention and treatment is still very minimal, if any. The pathogenetic mechanisms underlying both of these ocular complications of diabetes are considered multifactorial. The major factors considered to be involved in diabetic cataractogenesis are the hyperglycemia-induced excessive generation of free radicals and consequent oxidative stress as well as aberrations in tissue metabolism. Free radicals have also been implicated in the pathogenesis of diabetic retinopathy (DR). In addition, DR is considered to be associated with aberrations in several other cellular signaling pathways mediated via inflammatory intermediators, pigment epithelium-derived factor, various kinases, and Rho and Ras GTPases. Identification of pathways/molecules that can be targeted for modification by appropriate compounds is highly essential for the development of pharmacological therapy for these highly prevalent blinding diseases. In this special issue intended to present new research on diabetic cataracts and retinopathy, we have invited papers that address these issues.

The first four articles in this issue are focused on cataract formation in diabetes. The first one provides an overview of the pathogenesis of diabetic cataracts, clinical studies investigating the association between diabetes and the development of cataract, and current treatment of cataracts in the diabetic population. The anticataractogenic potential of aldose reductase inhibitors and antioxidants has also been discussed. The article by Petrash et al. focuses on the role of aldo-ketoreductases in the eye. The expression profiles of the AKR1B1 and AKRB10 have been characterized in various human ocular tissues and their implications in the pathogenesis of diabetic cataract have been discussed. The next article examines the role of glyoxalase I in the inhibition of methylglyoxal advanced glycation end products-AGEs formation in lenses of transgenic mice overexpressing human glyoxalase I, and the fourth one reviews the pathophysiological characteristics of ocular complications in the Spontaneously Diabetic Torii rat such as the formation of cataracts which can become hypermature. It also suggests the suitability of this model for the study of DR. Subsequent articles in this issue focus on the pathogenesis of DR and examine possible targets for pharmacological intervention. The article by Daniel Ng reviews the genetic aspects of DR, and the next article identifies genes associated with DR among Mexican Americans from Starr County in Texas using genome-wide association study. The seventh paper examines the possibility of treatment with the angiotensin-converting enzyme inhibitor, trandolapril, for regression of DR, and the 8th paper discusses the role of toxic AGEs receptor in the pathogenesis of DR. This is followed by a review implicating Rho/ROCK (Rho Kinase) pathway in DR and suggests the possibility of this pathway being a target for therapeutic intervention. The article by Wu et al. investigates the role of extracellular signal-regulated kinase 5 (ERK5) mediated signaling in modulating glucose-induced expression of VEGF. The final paper in this issue compares NADPH oxidase versus mitochondria-derived reactive oxygen species in glucose-induced apoptosis of pericytes in early DR.

This special issue will therefore provide the readers an insight into some of the latest research being pursued to elucidate the mechanisms underlying DR and cataracts. It also presents some interesting targets which could be manipulated pharmacologically for potential clinical use.

**Kavita R. Hegde** **Kavita R. Hegde** **Renu A. Kowluru** **Renu A. Kowluru** **Susanne Mohr** **Susanne Mohr** **Ram H. Nagaraj** **Ram H. Nagaraj** **J. Mark Petrash** **J. Mark Petrash**

